# Cardiac Sympathetic Hyperactivity after Chemotherapy: Early Sign of Cardiotoxicity?

**DOI:** 10.5935/abc.20150075

**Published:** 2015-09

**Authors:** Sarita Lígia Pessoa de Melo Machado Guimarães, Simone Cristina Soares Brandão, Luciana Raposo Andrade, Rafael José Coelho Maia, Brivaldo Markman Filho

**Affiliations:** 1 Pós-Graduação em Ciências da Saúde da Universidade Federal de Pernambuco (PGCS-UFPE), Recife, PE – Brazil; 2 Hospital Agamenon Magalhães (HAM), Recife, PE – Brazil; 3 Hospital Santa Joana, Recife, PE – Brazil

**Keywords:** Drug Therapy, Drug-Related Side Effects and Adverse Reactions, Sympathetic Nervous System, Breast Neoplasms

## Abstract

**Background:**

Chemotherapy with anthracyclines and trastuzumab can cause cardiotoxicity. Alteration of cardiac adrenergic function assessed by metaiodobenzylguanidine labeled with iodine-123 (^123^I-mIBG) seems to precede the drop in left ventricular ejection fraction.

**Objective:**

To evaluate and to compare the presence of cardiovascular abnormalities among patients with breast cancer undergoing chemotherapy with anthracyclines and trastuzumab, and only with anthracycline.

**Methods:**

Patients with breast cancer were analyzed clinical, laboratory, electrocardiographic and echocardiographic and cardiac sympathetic activity. In scintigraphic images, the ratio of ^123^I-mIBG uptake between the heart and mediastinum, and the washout rate were calculated. The variables were compared between patients who received anthracyclines and trastuzumab (Group 1) and only anthracyclines (Group 2).

**Results:**

Twenty patients, with mean age 57 ± 14 years, were studied. The mean left ventricular ejection fraction by echocardiography was 67.8 ± 4.0%. Mean washout rate was 28.39 ± 9.23% and the ratio of ^123^I-mIBG uptake between the heart and mediastinum was 2.07 ± 0.28. Of the patients, 82% showed an increased in washout rate, and the ratio of ^123^I-mIBG uptake between the heart and mediastinum decreased in 25%. Concerning the groups, the mean washout rate of Group 1 was 32.68 ± 9.30% and of Group 2 was 24.56 ± 7.72% (p = 0,06). The ratio of ^123^I-mIBG uptake between the heart and mediastinum was normal in all patients in Group 2, however, the Group 1, showed 50% the ratio of ^123^I-mIBG uptake between the heart and mediastinum ≤ 1.8 (p = 0.02).

**Conclusion:**

In women with breast cancer undergoing chemotherapy, assessment of cardiac sympathetic activity with ^123^I-mIBG appears to be an early marker of cardiotoxicity. The combination of chemotherapy showed higher risk of cardiac adrenergic hyperactivity.

## Introduction

Breast cancer is the most common neoplasm in women and its incidence is increasing. As the population ages, additional risk factors should be included, such as cardiovascular disease^1,2^.

Chemotherapy, which improves survival, is crucial for the treatment of several cancers, but has potential risks for toxicity^3^. For several years, anthracyclines have been used in the treatment of breast cancer in its different stages^4^. Its use has shown to be very advantageous^5,6^.

Approximately 20 to 30% of patients with breast cancer have tumors with amplification of the HER2 / neu gene^7^, of which expression results in a lower response to chemotherapy, due to rapid growth of the malignant cells^6,7^. However, HER2-positive breast cancer responds favorably to trastuzumab, a monoclonal antibody that targets this receptor^8^.

Both anthracyclines and trastuzumab are associated with myocardial injury^1,3^. Cardiotoxicity is a clinically silent complication that can occur in up to 27% of patients undergoing chemotherapy^9^.

Routine cardiologic assessment in chemotherapy users is performed by assessing symptoms and through serial electrocardiograms and echocardiograms. Once the decrease in ventricular function is detected, therapeutic measures may be necessary, including the interruption of chemotherapy^1^.

Autonomic innervation plays a key role in regulating heart rate, myocardial function and myocardial blood flow. Its impairment usually means the presence of disease and may precede alterations in myocardial contractility and also reflect disease severity^10^. The assessment of cardiac autonomic function can be performed non-invasively using scintigraphic imaging with specific radiotracers.

To identify new ways to perform early cardiovascular risk assessment in patients treated with potentially cardiotoxic drugs is a challenge. The aim is to prevent chemotherapy interruption, while cardioprotective drugs, such as beta-blockers and angiotensin inhibitors are started early, thus preventing further damage and progression of myocyte injury.

The objective of this study was to evaluate and compare the presence of cardiovascular alterations in patients with breast cancer undergoing chemotherapy with anthracyclines and trastuzumab, or only anthracyclines.

## Methods

### Study population

This study was a case series that consecutively included 20 female patients with breast cancer, aged ≥ 18 years. The patients were further divided into two groups: Group 1 patients that were treated with anthracyclines associated with trastuzumab; and Group 2 patients that were treated with anthracyclines only. Patients with known heart disease or heart failure symptoms were excluded. None of the patient had Parkinson's disease or any other known neurological disease, considering these conditions may cause changes in cardiac sympathetic activity with ^123^I-mIBG^11^.

Group 2 patients were studied after treatment with anthracyclines and those from Group 1, after treatment with anthracyclines and during treatment with trastuzumab, having already received at least two infusions.

The study was approved by the Research Ethics Committee (protocol number CAAE-0001.0.236.000-11). All patients agreed to participate and signed the free and informed consent form.

### Study site

From November 2010 to May 2012, of all patients referred from the Oncology service to the Cardiology Clinic for cardiovascular risk assessment that met the inclusion criteria were invited to participate in this study.

### Study variables

All patients were submitted to clinical history, physical examination, electrocardiogram, echocardiography and cardiac scintigraphy ^123^I-mIBG. Clinical and laboratory data, such as age, weight, height, body mass index, blood pressure measurement, medications being used, diabetes mellitus, smoking status, total cholesterol, High-Density Lipoprotein-cholesterol (HDL-c) and Framingham risk score, were obtained and recorded in a specific form.

Framingham risk score was calculated using data from the clinical history (age in years, female gender, known diabetic and smoker), physical examination (systolic blood pressure at rest measured in mmHg) and laboratory tests (values of total cholesterol, HDL-c and glucose measurements)^12^.

The electrocardiogram was considered normal or altered, according to the guidelines of the Brazilian Society of Cardiology on Analysis and Release of Electrocardiographic Reports^13^.

Doppler echocardiography was performed in a Philips HD7 device, serial number CI51100623, manufactured in September 2010 (Diagnostic Ultrasound System, Bothell, Washington, United States). The left ventricular ejection fraction (LVEF) was calculated using the Teichholz method. The examinations were performed as recommended by the Department of Cardiovascular Imaging of the Brazilian Society of Cardiology^14^.

**Figure 1:**
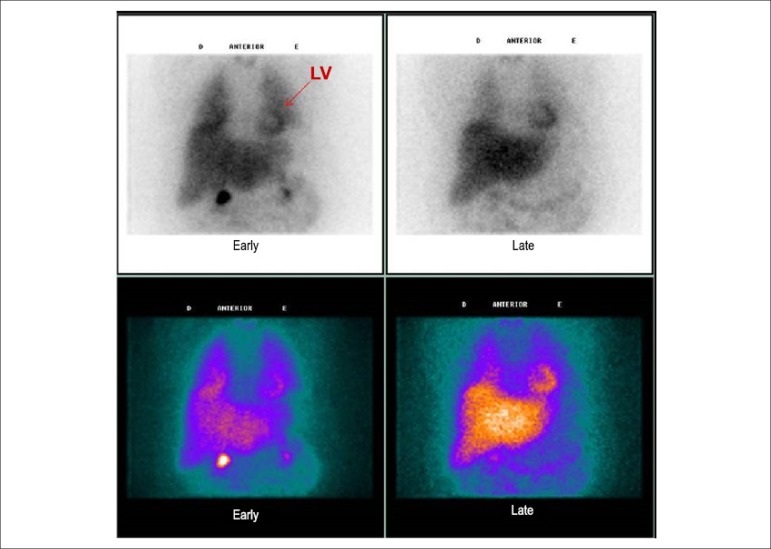
Cardiac scintigraphy with ^123^I-mIBG of a study patient at the anterior chest view 10 minutes (early) and 4 hours (late) after injection of ^123^I-mIBG. Upper panel: black-and-white images; lower panel: color images. LV: left ventricle.

For the cardiac scintigraphy ^123^I-MIBG, planar images of the chest were obtained, approximately 15 minutes (early images) and 4 hours (delayed images) after intravenous injection of 185 MBq (5 mCi) of ^123^I-mIBG (Figure 1). Images were acquired in a tomographic gamma camera with two detectors, an Infinia Hawkeye-4 model (General Electric Medical Systems, Milwaukee, Wisconsin, United States), with a collimator for low energy and high resolution. The energy photopeak was centered at 159 keV with a window of 20% and matrix of 128 x 128. Static acquisition of 10 minutes was performed using the anterior chest view, in the early and late stages, after the radioisotope injection. A Region of Interest (ROI) was manually drawn on the heart (H) and over a nine-pixel area in the upper mediastinum (M) and the mean number of counts was obtained for each of these regions.

The heart/mediastinum rate (H/MR) and the washout rate in percentage (WR) were calculated using the early and late images. The WR was considered as the percentage of decrease in cardiac activity between the early and late images within the left ventricle area. H/MR values ≤ 1.8 and WR > 19% were considered abnormal, i.e., indicative of adrenergic hyperactivity^15^. It was not possible to calculate the WR in two patients from Group 1 and one from Group 2 due to technical problems on the day the images were obtained.

All patients received potassium iodide syrup (500 mg) by mouth about 1 hour before administration of ^123^I-MIBG for the blocking of the thyroid gland.

### Statistical Analysis

The sample of 20 patients was a convenience one. Continuous quantitative variables were expressed as mean and standard deviation. Mann-Whitney U-test was used to compare continuous variables between the two independent groups. Categorical variables are shown as frequencies and percentages. The canonical test was used to compare variables expressed in percentage between the groups. Spearman’s correlation was used in Group 1 to evaluate the correlation between the number of trastuzumab cycles and the mIBG scintigraphy variables. Statistical significance was set at 5% (p < 0.05). The Statistical Package for Social Sciences (SPSS) software, version 13.0 (SPSS Inc., Chicago, Illinois, United States) was used for the statistical analysis.

## Results

### Clinical, electrocardiographic and echocardiographic characteristics

The mean age of the 20 patients was 57.3 ± 13.8 years. Body mass index was 27.9 ± 4.0, indicating an overweight population. The mean Framingham risk score was 5.7%, which characterizes a subgroup with low risk for coronary events in 10 years. No significant differences were observed regarding the clinical variables in the groups.

Only two patients showed ventricular repolarization alterations on the electrocardiography. On the echocardiography, the mean ejection fraction was 67.8 ± 4.0%. All patients had LVEF> 55%, i.e., normal ventricular function (Table 1). The left atrium and left ventricle cavity diameters were within normal parameters. No significant differences were observed regarding the echocardiographic variables between the groups.

**Table 1:** Descriptive statistics of Doppler echocardiography measurements of the 20 patients

Variable	Mean	SD	Minimum	Maximum
LA (cm)	3,180	0,3365	2,60	3,80
LVDD (cm)	4,670	0,3342	4,00	5,60
LVSD (cm)	2,945	0,3137	2,60	3,90
E/A	1,049	0,3960	0,50	1,68
LVEF	67,850	4,0167	57,00	75,00

SD: standard deviation; LA: left atrium; LVDD: left ventricular diastolic diameter; LVSD: left ventricular systolic diameter; E/A: ratio between the maximum velocities of E and A waves on transmitral spectral Doppler; LVEF: left ventricular ejection fraction.

### Cardiac sympathetic activity assessment

Regarding the assessment of cardiac sympathetic activity, the mean WR in Group 1 was 32.68 ± 9.30%, and in Group 2, 24.56 ± 7.72%, p = 0.06 (Figure 1). The early mean H/MR was 1.94 ± 0.28 in Group 1 and 2.20 ± 0.23 in Group 2 (p = 0.03).

About 82% of the assessed patients showed an increased WR (normal value < 19%) and 25% had a decreased early H/MR, i.e. ≤ 1.8. However, in Group 1, WR was normal in only one patient, and in five, this index was > 30%, as shown in Table 2. In Group 2, 44% of patients had a normal or slightly altered WR, and only two had WR > 30% (Table 3). The H/MR was normal in all Group 2 patients; however, in Group 1, 50% had reduced H/MR (p = 0.02).

**Table 2:** Clinical characteristics and test results of patients from group 1 treated with anthracyclines and trastuzumab

Patient	Age (years)	Cycles	Framingham's Risk (%)	SAH	DM	ECG	DD	ACEI/BB	WR (%)	H/MR
1	68	2	4	-	-	-	Yes	-	28	2.3
2	58	8	8	-	-	-	-	-	32.6	1.7
3	44	6	1	-	-	-	-	-	30.4	2.0
4	67	14	2	-	-	-	-	-	15.7	1.7
5	51	17	1	-	-	-	Yes	-	39.9	2.3
6	80	7	8	Yes	Yes	-	Yes	Yes	46.5	1.7
7	76	18	17	Yes	-	-	-	Yes	38.8	1.6
8	34	10	1	-	-	-	-	-	29.6	2.3
9	42	3	4	-	-	-	-	-	*	1.8
10	32	12	1	-	-	-	-	-	*	2.0

* Technical problems prevented the calculation of CR in two patients. SAH: systemic arterial hypertension; DM: diabetes mellitus; ECG: electrocardiogram; DD: diastolic dysfunction verified by echocardiography; ACEI: angiotensin-converting enzyme inhibitors; BB: beta-blockers; WR: washout rate of mIBG; H/MR: heart/ mediastinum rate of mIBG.

**Table 3:** Clinical characteristics and test results of patients from group 2 treated with anthracyclines only

Patient	Age (years)	Framingham's Risk (%)	SAH	DM	ECG	DD	ACEI/BB	WR (%)	H/MR
1	44	<1						25.9	2.0
2	63	1						21.2	2.0
3	50	1						27.5	2.2
4	60	1				Yes		34.1	2.3
5	77	22	Yes			Yes	Yes	*	2.1
6	65	11	Yes		Yes		Yes	19.3	2.2
7	61	11	Yes			Yes	Outro	29.8	2.2
8	53	3						14.7	2.4
9	69	13	Yes				Yes	34.6	1.9
10	52	7			Yes	Yes		14.0	2.7

* Technical problems prevented the calculation of CR in two patients. SAH: systemic arterial hypertension; DM: diabetes mellitus; ECG: electrocardiogram; DD: diastolic dysfunction verified by echocardiography; ACEI: angiotensin-converting enzyme inhibitors; BB: beta-blockers; WR: washout rate of mIBG; H/MR: heart/ mediastinum rate of mIBG

### Correlation between the number of trastuzumab cycles and cardiac sympathetic activity

Considering the variable number of cycles of trastuzumab administered to patients in Group 1, we performed an analysis of the scintigraphy measurements in patients that had fewer than eight cycles (Rho) and the ones that had more than eight cycles (R2). There seems to be a positive correlation between the WR and the number of cycles (rho = 0.47; p = 0.06), as well as a negative one between the early H/MR and the number of trastuzumab cycles (rho = -0.40; p = 0.08).

## Discussion

This was the first Brazilian study that used cardiac scintigraphy performed with ^123^I-mIBG to identify early cardiac injury after chemotherapy, as the autonomic dysfunction may precede ventricular dysfunction and, consequently, the drop in left ventricular ejection fraction^15^.

Decreased cardiac output in HF activates a series of adaptations in an attempt to maintain cardiovascular homeostasis. One of the most important is the activation of the sympathetic nervous system (adrenergic), which occurs in the beginning of HF^16^. The results of this study suggest an association between anthracycline use and increased cardiac sympathetic activity, whereas the addition of trastuzumab resulted in an even greater hyperactivity adrenergic. It is noteworthy, as shown before, that none of these patients had classic clinical and/or echocardiographic signs of HF and only two had electrocardiographic alterations. Thus, the alteration in cardiac sympathetic activity assessed by ^123^I-mIBG seemed to precede clinical signs of HF and the decrease in LVEF. It may be the initial trigger for the development of symptomatic heart failure, if that neurohormonal dysfunction progresses or worsens.

When we analyze our results, we observe that, in both groups, most patients showed an accelerated washout rate of ^123^I-mIBG, i.e., > 19%. The mean WR was statistically higher in Group 1 than in Group 2. When we analyze the early H/MR in Group 1, five patients (50%) had this index ≤ 1.8, whereas in Group 2, none of the patients had a decreased index. Moreover, the mean H/MR was statistically lower in Group 1.

Another interesting finding was the correlation between the number of trastuzumab cycles and the cardiac sympathetic activity assessment. A trend towards a positive correlation between the value of WR, and a negative correlation between H/MR and the number of received cycles was observed. This seems to indicate that, the more cycles received, there seems to be a major change in cardiac innervation and sympathetic activity.

Carrió et al.^17^ identified an abnormal uptake of ^123^I-mIBG in patients that received anthracyclines and also, the H/MR was lower as the cumulative dose of this medication progressed. Jacobson et al.^18^ found that HF patients with H/MR < 1.6 had increased cardiovascular risk.

Systolic dysfunction, after exposure to cardiotoxic agents, is usually an irreversible, progressive and lethal condition^19^. The development of HF occurs in up to 27% of women that receive the anthracycline-trastuzumab combination and, therefore, a careful clinical management of these patients is recommended^5^. New echocardiographic modalities, such as the use of tissue Doppler, regional strain and strain rate, may increase the method sensitivity to detect subclinical ventricular dysfunction, as well as new biochemical markers such as troponins and BNP^19^. A recent Brazilian study^20^, which included 51 patients treated with trastuzumab for advanced HER2-positive breast cancer, has shown that at the third month of treatment, clinical and biochemical data (troponin and NT-proBNP measurements) were not statistically different at the beginning and after 3 months of treatment with trastuzumab. However, a statistically significant difference was observed between the E/e' ratio at the beginning and after the third month of follow-up, which was closely related to a decrease in myocardial e’ velocity, as assessed by tissue Doppler on echocardiography.

In this study, a more detailed analysis of diastolic function by tissue Doppler was not part of our objectives. The diastolic function analysis of our sample was obtained by echocardiography, using the maximum A/E velocity ratio by transmitral spectral Doppler, and the evaluation was performed only at the end of chemotherapy with anthracycline and during treatment with trastuzumab. Thus, there were no differences between the groups regarding the frequency of diastolic dysfunction. Three patients from Group 1 and four from Group 2 showed myocardial relaxation alterations, but there was no association with the degree of cardiac adrenergic hyperactivity by ^123^I-mIBG.

Studies with a larger number of patients using these more sensitive methods, together with data on cardiac scintigraphy with ^123^I-mIBG are useful to clarify the results of this study, as well as the long-term follow-up of these patients with exacerbated cardiac sympathetic activity.

### Study Limitations

It is important to emphasize that the elderly, hypertensive and/or diabetic patients may have cardiac sympathetic dysfunction as part of the underlying disease. On the other hand, patients using drugs with cardioprotective potential, such as Angiotensin-Converting Enzyme inhibitors (ACEI) and beta-blockers, may progress with improved cardiac sympathetic function and systolic dysfunction^7,21^. In this study, four patients from Group 2 were hypertensive and three patients were receiving cardioprotective drugs. In Group 1, two patients were hypertensive, one was diabetic and two were receiving cardioprotective medications. These factors must have influenced sympathetic activity assessment; however, as they were not statistically different between the groups, the comparative analysis of cardiac sympathetic activity does not seem to have been influenced.

Another limitation of the present study was the small sample size. Although the data collection time was relatively long, oncologists were not used to referring patients for cardiac assessment. This interaction between these two specialties should be encouraged, considering the cardiotoxic potential of these drugs and the increased survival of cancer patients, which may be cured or live peaceably with the cancer, but who may die prematurely from heart disease secondary to chemotherapy, if not diagnosed and treated early. The lack of inclusion of a control group also decreases the certainty of these results. However, this study seems to corroborate findings from a previously published study^6^ and emphasize the need to confirm, with larger studies, the value of cardiac sympathetic activity assessment with ^123^I-mIBG in the follow-up of patients undergoing chemotherapy with potentially cardiotoxic drugs.

## Conclusion

In women with breast cancer submitted to chemotherapy with potentially cardiotoxic drugs, assessment of cardiac sympathetic activity with ^123^I-mIBG can be an early marker of cardiac injury. The use of anthracycline derivatives with trastuzumab resulted in higher frequency and intensity of cardiac adrenergic hyperactivity. Studies with larger samples comparing the assessment of cardiac sympathetic activity with mIBG before and after treatment need to be performed to verify these findings.
